# Effects of Hepatocyte CD14 Upregulation during Cholestasis on Endotoxin Sensitivity

**DOI:** 10.1371/journal.pone.0034903

**Published:** 2012-04-12

**Authors:** Ming-Huei Chou, Jiin-Haur Chuang, Hock-Liew Eng, Po-Chin Tsai, Chih-Sung Hsieh, Hsiang-Chun Liu, Chiou-Huey Wang, Chih-Yun Lin, Tsun-Mei Lin

**Affiliations:** 1 Institute of Basic Medical Sciences, National Chang Kung University, Tainan, Taiwan; 2 Graduate Institute of Clinical Medical Sciences, Chang Gung University, Kaohsiung, Taiwan; 3 Department of Surgery, Kaohsiung Chang Gung Memorial Hospital and Chang Gung University College of Medicine, Kaohsiung, Taiwan; 4 Department of Pathology, Kaohsiung Chang Gung Memorial Hospital and Chang Gung University College of Medicine, Kaohsiung, Taiwan; 5 Department of Medical Research, PingTung Christian Hospital, PingTung, Taiwan; 6 Departmentof Laboratory Medicine, E-DA Hospital/I-SHOU University, Kaohsiung, Taiwan; 7 Division of Hepato-gastroenterology, Kaohsiung Chang Gung Memorial Hospital and Chang Gung University College of Medicine, Kaohsiung, Taiwan; 8 Department of Medical Research, E-DA Hospital/I-SHOU University, Kaohsiung, Taiwan; The Scripps Research Institute, United States of America

## Abstract

Cholestasis is frequently related to endotoxemia and inflammatory response. Our previous investigation revealed a significant increase in plasma endotoxin and CD14 levels during biliary atresia. We therefore propose that lipopolysacharides (LPS) may stimulate CD14 production in liver cells and promote the removal of endotoxins. The aims of this study are to test the hypothesis that CD14 is upregulated by LPS and investigate the pathophysiological role of CD14 production during cholestasis. Using Western blotting, qRT-PCR, and promoter activity assay, we demonstrated that LPS was associated with a significant increase in CD14 and MD2 protein and mRNA expression and CD14 promoter activity in C9 rat hepatocytes but not in the HSC-T6 hepatic stellate cell line *in vitro*. To correlate CD14 expression and endotoxin sensitivity, *in vivo* biliary LPS administration was performed on rats two weeks after they were subjected to bile duct ligation (BDL) or a sham operation. CD14 expression and endotoxin levels were found to significantly increase after LPS administration in BDL rats. These returned to basal levels after 24 h. In contrast, although endotoxin levels were increased in sham-operated rats given LPS, no increase in CD14 expression was observed. However, mortality within 24 h was more frequent in the BDL animals than in the sham-operated group. In conclusion, cholestasis and LPS stimulation were here found to upregulate hepatic CD14 expression, which may have led to increased endotoxin sensitivity and host proinflammatory reactions, causing organ failure and death in BDL rats.

## Introduction

Cholestasis, impairment of bile outflow, occurs in a wide variety of human liver diseases [1]–[3]. Endotoxemia, which is a frequent complication of cholestasis, can also be caused by a decrease in bile flow promoting bacterial translocation from the gut [4]. Lipopolysacchaides (LPS), which make up parts of the outer membranes of Gram negative enterobacteria, provoke proinflammatory responses and cause hepatocellular injury by promoting liver dysfunction and fibrogenesis, ultimately leading to liver failure [5]. Using an animal model of bile duct ligation (BDL), it has been shown that low levels of intestinal bile acids may account for the high frequency of endotoxemia in the portal and peripheral blood during cholestasis [6]. The liver is the major organ downstream of the gut and is responsible for LPS clearance by both parachymal cells (hepatocytes) and nonparachymal cells (hepatic stellate cells and Kupffer cells) [7]. Kupffer cells have been found to modulate LPS for rapid internalization within hepatocytes to clear through bile flow and prevent systemic distribution and widespread inflammatory reactions during endotoxemia [7], [8]. In previous reports, LPS and bacterial clearance from the liver were found to be reduced in BDL compared with sham-operated animals through the impairment of phagocytic ability and an inability to kill intracellular bacteria in Kupffer cells [9]–[11]. However, the mechanisms underlying the impaired endotoxin clearance capacity of hepatocytes during cholesatasis are not clearly defined.

CD14 is thought to be the important LPS receptor and exists in membrane (mCD14) and soluble (sCD14) forms [12], [13]. Expressed on the surface of monocytes, macrophages and immune cells, mCD14 is a 50–55 KDa receptor linked to the cell surface by a glycosyl-phosphatidyl inositol (GPI) anchor. Soluble CD14 can be released by shedding via protease-dependent or independent pathways, or secreted directly after synthesis [14]. Human hepatocytes were demonstrated to produce CD14 by a mechanism similar to producing acute-phase proteins [10], [11]. In liver tissue, Kupffer cells and sinusoidal endothelial cells express mCD14, while hepatocytes are the main producers of sCD14 in plasma [15]. Billiar et al. demonstrated that CD14, TLR4, and MD2 form a multi-receptor complex within the lipid rafts of hepatocytes for LPS uptake and signal activation [16]. Our previous investigation revealed a significant increase in plasma endotoxin and sCD14 levels during biliary atresia. In addition, we also found that both endotoxin and CD14 levels were significantly increased in the liver tissues of rats following BDL [17]. We therefore propose that LPS may stimulate CD14 production in liver cells during the early stage of biliary atresia, promoting endotoxin removal, and that endotoxin signaling likely induces liver injury and impairs CD14 synthesis during the later stages.

Although mouse and human hepatocytes have also shown increased expression of CD14 during endotoxemia, CD14 production in the liver and the subsequent effects on endotoxin-induced liver injury during obstructive jaundice remain unclear [15], [18]. The aims of this study are to test the hypothesis that CD14 is upregulated by LPS for endotoxin clearance and to investigate the pathophysiological mechanisms and roles of CD14 production during cholestasis. We evaluate the *in vitro* effect of LPS on TLR4, CD14, and MD2 expression in hepatocytes and a hepatic stellate cell line and on endotoxin sensitivity during cholestasis by BDL animals.

## Results

### Effects of LPS treatment on CD14 and MD2 expression in rat hepatocytes

**Figure 1:**
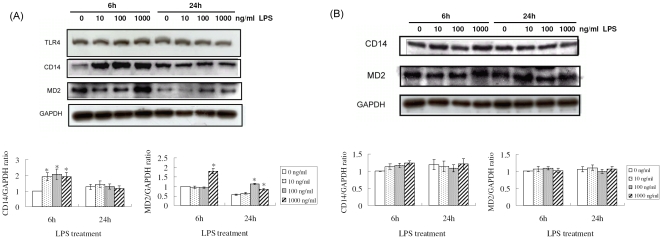
Western blot analysis of TLR4, CD14, and MD2 protein. TLR4, CD14, and MD2 levels analyzed in (A) C9 rat hepatocytes and (B) HSC-T6 cells after LPS treatment. Total protein was extracted from C9 rat hepatocytes and HSC-T6 cell lines at 6 h and 24 h after stimulation with 10, 100, or 1,000 ng/mL LPS. Protein extracts separated by SDS-PAGE were immunoblotted for TLR4, CD14, and MD2 and β-actin as loading controls. The bar graph depicts the CD14 and MD2 vs β-actin density ratio by densitometer determination.* indicates *P*<0.05 relative to control cells (without LPS treatment). All results were obtained from at least three separate experiments.

To evaluate TLR4, CD14, and MD2 protein expression in liver cells after LPS treatment, Western blot analysis was performed on protein from C9 rat hepatocytes and HSC-T6 cells stimulated with various concentrations of LPS. Lysate from C9 rat hepatocytes treated with LPS for 6 h contained 2 fold more CD14 than control cells and the CD14 levels declined to levels comparable to baseline 24 h after treatment. MD2 protein was significantly increased only after treatment with 1,000 mg LPS, but no difference in TLR4 protein levels was observed (Figure 1A). In contrast, for HSC-T6 cells, CD14, and MD2 protein expression showed no significant fluctuations after 6 h or 24 h of LPS treatment (Figure 1B), and barely a trace of TLR4 was detected at any time.

### Effects of LPS treatment on mRNA expression of hepatocyte CD14 and MD2

**Figure 2:**
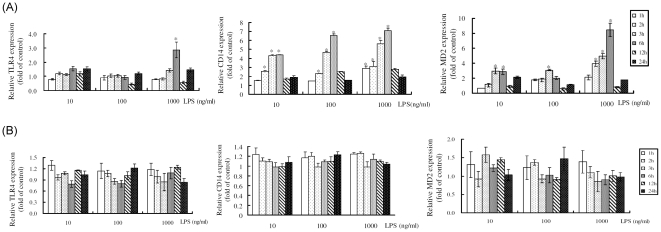
Kinetic changes in TLR4, CD14, and MD2 mRNA expression detected by qRT- PCR after LPS treatment. Total RNA isolated from (A) C9 rat hepatocytes and (B) HSC-T6 cells at 1, 2, 3, 6, 12, and 24 h after 10–1,000 ng/mL LPS treatment. TLR4, CD14, and MD2 mRNA expression was normalized using the mRNA of housekeeping gene GAPDH. The expression of TLR4, CD14, and MD2 mRNA expression is shown relative to that of the time-matched control cells (without LPS treatment). Data are expressed as mean±SE of three separate experiments.* indicates *P*<0.05 relative to time-matched control cells.

C9 rat hepatocytes and HSC-T6 cells were incubated with different concentrations of LPS (10 ng/mL, 100 ng/mL, and 1,000 ng/mL) and total RNA was extracted from harvested cells at different points in time. For C9 hepatocytes, quantitative RT-PCR analysis showed the expression of CD14 mRNA was increased at 2 h, 3 h, and 6 h after treatment with 10 ng/ml, 100 ng/ml, and 1,000 ng/mL of LPS, showing a remarkable 7.5-fold increase at 6 h after administration of 1,000 ng/mL LPS. The expression of MD2 mRNA was increased at 2 h, 3 h, and 6 h after treatment with 1,000 ng/mL of LPS. However, TLR4 mRNA levels did not significantly change (Figure 2A). In contrast, TLR4, CD14, and MD2 mRNA expressions did not significantly change in HSC-T6 hepatic stellate cells at any point in time after LPS treatment (Figure 2B)

### Effects of LPS treatment on CD14 promoter activity induction in rat hepatocytes and hepatic stellate cells

**Figure 3:**
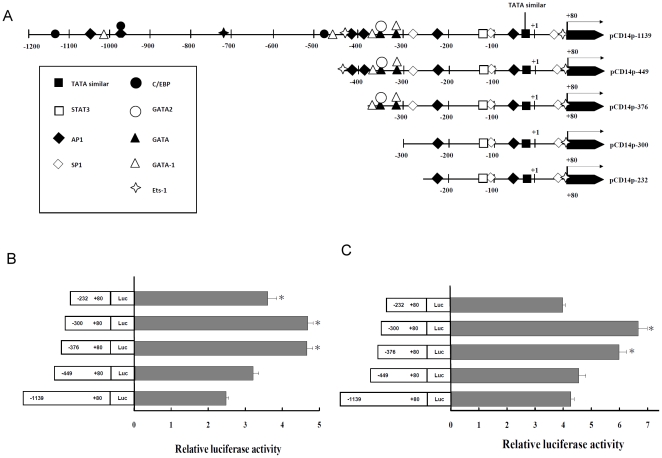
Deletional analysis of the CD14 promoter. (A) Schematic representation of CD14 deletional constructs. A series of 5′-deletional segments was generated by inserting into the multiple cloning sites upstream the luciferase reporter gene in a promoterless and enhancerless vector, pGL3. Transient transfection in (B) C9 rat hepatocytes and (C) HSC-T6 cells. Cells were transiently transfected with the constructs described in (A). Luciferase activity was measured 24 h after transfection. The promoter activity of each construct is calculated relative to the activity of the pGL3 vector. Data shown are mean±S.E. of five independent experiments with triplicate samples used in each experiment (*indicates *P*<0.05 relative to pCD14p−1139).

After sequence analysis revealed some putative binding motifs within −1139 bp to +80 bp upstream of the transcription initiation site of the CD14 promoter region (Figure 3A), serially deleted CD14 promoter-driven luciferase reporter gene constructs at 449, 376, 300, and 232 bp upstream of the transcription initiation site and control pGL3 basic-LUC vector were transfected into C9 rat hepatocytes and HSC-T6 cells. The relative luciferase expression ratios of each reporter gene construct (Figure 3B and 3C) revealed similar patterns and promoter activities in C9 rat hepatocytes and HSC-T6 cells. Deletion in the region from −449 to −376 caused increased transcription of the reporter gene, while deletion in the region from −300 to −232 caused a decrease in promoter activity. These findings suggest that negative and positive regulation elements may exist within the −449 to −376 and −300 to −232 regions, respectively. These data reveal the presence of functional promoter activity, especially an activation domain between −376 and +80 bp in the proximal promoter of the CD14 gene in both cell lines (Figures 3B, 3C).

**Figure 4:**
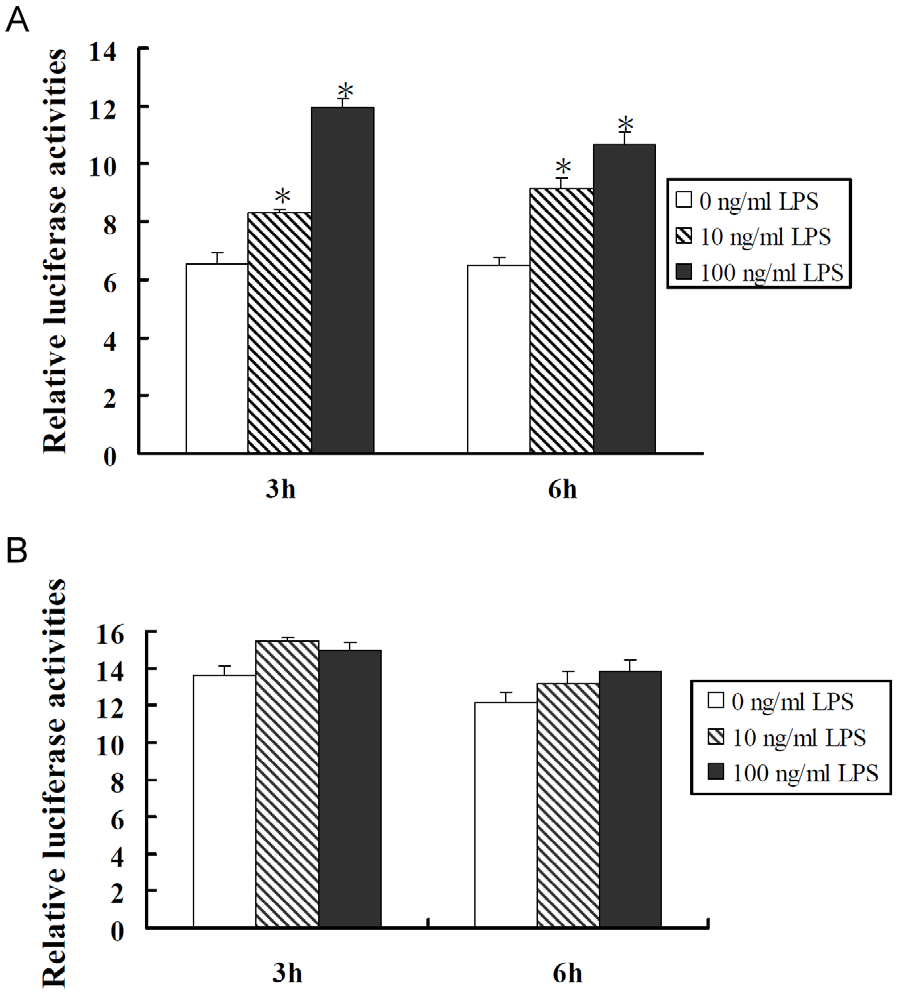
Effect of LPS on CD14 promoter activity. (A) C9 rat hepatocytes and (B) HSC-T6 cells were transfected with plasmid of pCD14p-376. After transfection for 24 h, cells were treated with LPS (10 and 100 ng/mL) for 3 and 6 h. Cells were harvested and luciferase activity was measured. Data are expressed as mean±SE of the luciferase activities relative to the pGL3 expression in the respective control groups. * indicates *P*<0.05 versus time-matched values for the control cells (without LPS treatment).

We compared the induction activity of LPS on the construct with −376 to +80 region of CD14 promoter region in C9 rat hepatocytes and HSC-T6 cells. Mean luciferase values relative to the pGL-3 basic vector are given in Figures 4A and 4B. A dose-dependent increase in the relative luciferase activities after 3 h and 6 h of LPS treatment was observed in C9 rat hepatocytes. However, there was no significant difference in the reporter activity of CD14-376 promoter construct in HSC-T6 cells with LPS treatment.

### Changes in biochemical parameters after LPS treatment in BDL rats

Biochemical parameters after either sham operation or bile duct ligation lasting 2 weeks are summarized in Table 1. In sham-operated rats, levels of AST, ALT, and T-Bil (135±56.2 U/I, 51±8.8 U/I, 0.09±0.01 mg/dL, respectively) were similar to those of control rats. Rats receiving BDL showed significantly increased basal levels of AST, ALT, and T-Bil (670±191 U/I, 155±43 U/I, 7.4±1.7 mg/dL, respectively). After 0.5 mg/kg LPS administration, levels of AST, ALT, and T-Bil were all significantly elevated at 3 h (2084±430 U/I, 486±88 U/I, 11.13±0.8 mg/dL, respectively) but only AST and ALT (2122±488 U/I, 413±116 U/I; respectively) remained higher than in the untreated group after 24 h of LPS treatment. AST and ALT levels were also significantly higher in sham-operated rats than the corresponding untreated groups after LPS administration lasting 24 h.

**Table 1:** Routine parameters of experimental animals.

	Survival/total No	AST (U/l)	ALT (U/l)	T Bilirubin (mg/dl)
Control	6/6	97±7.99	39±1.61	0.02±0.02
Sham-operated	6/6	135±56.2	51±8.76	0.09±0.01
Sham-LPS 3 h	6/6	200±32.7	73±13.5	0.12±0.02
Sham-LPS 24 h	5/6	399.0±73.0†	120±28.0†	0.18±0.08
BDL	6/6	670.2±191*	155±43.5*	7.42±1.67*
BDL-LPS 3 h	10/10	2084±430* †	486±88.0* †	11.13±0.83* †
BDL-LPS 24 h	5/10	2122±488* †	413±116* †	6.95±0.49*

Data represent means ±SE; BDL, bile duct ligation; LPS 3 h, rats sacrificed 3 h after 0.5 mg/kg LPS administration; LPS 24 h, rats sacrificed 24 h after 0.5 mg/kg LPS administration. AST, aspartate aminotransferase; ALT, alanine aminotransferase ; T-Bilirubin, total bilirubin.

* *p<0.05* versus time-matched values for the Sham-operated group;

† *p<0.05* versus values for the respective treatment control groups of BDL or Sham-operated rats. (by two-way ANOVA using Bonferroni's post hoc test).

**Figure 5:**
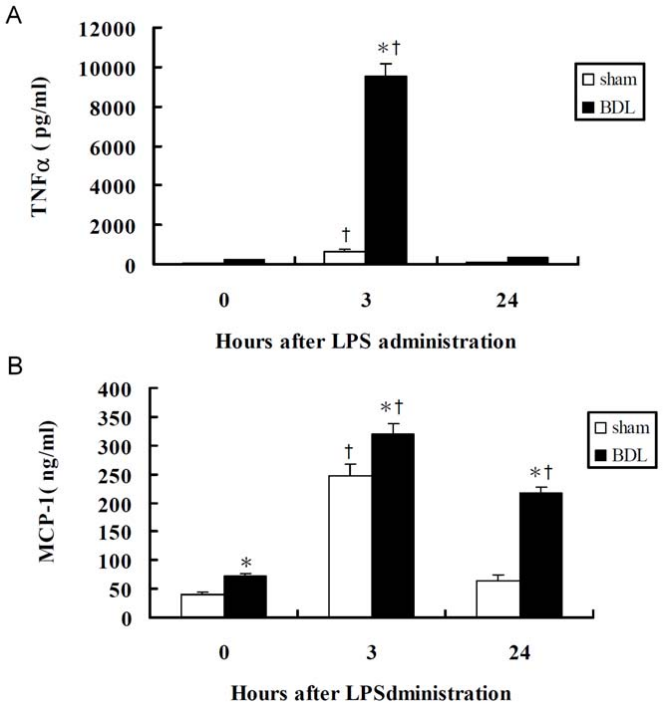
Effects of LPS treatment on BDL and sham-operated rats induced cytokine production. Plasma (A) TNFα and (B) MCP-1 were measured after 0.5 mg/kg LPS administration. All values are averages of 5–10 rates. Data represent mean±SE. * indicated *P*<0.05 versus time-matched values for the Sham-operated group; *^†^* indicates *P*<0.05 versus values for levels in untreated groups (0 h) of BDL or Sham-operated rats (as determined by two-way ANOVA using Bonferroni's post hoc test).

The plasma TNFα levels were nearly undetectable in the sham-operated and BDL groups but were approximately 14 times higher in BDL rats than in sham-operated rats at 3 h after 0.5 mg/kg LPS administration (9507.0±675.2 pg/ml vs 658.8±107.3 pg/ml, *P*<0.001) (Figure 5A). Baseline plasma MCP-1 levels were significantly lower in the sham-operated group than in the BDL group (41.17±3.96 pg/ml vs 73.33±2.99 pg/ml, *P* = 0.004). Three hours after biliary LPS (0.5 mg/kg) administration, plasma MCP-1 levels reached a maximum in both the sham-operated and BDL groups. In the sham-operated group, MCP-1 increased to 247.5±20.5 pg/ml after 3 h treatment and decreased to baseline at 24 h. In the BDL group, MCP-1 increased to 319.50±18.1 pg/ml at 3 h after treatment and was still high at 24 h (216.50±10.1 pg/ml) (Figure 5B).

**Figure 6:**
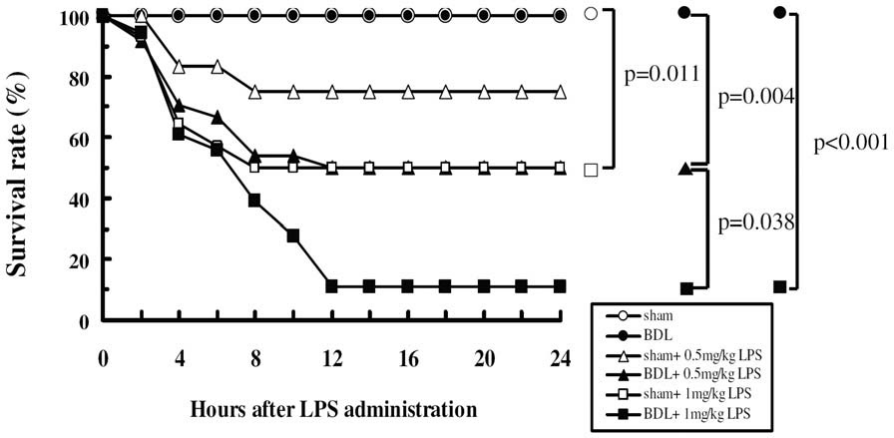
Survival curve for BDL and sham-operated rats after LPS infused into the biliary system within 24 h. Closed circles, BDL group (n = 12); opened circles, sham-operated group (n = 10); closed triangles, BDL with 0.5 mg/kg LPS administration (n = 24); opened triangles, sham-operated with 0.5 mg/kg LPS administration (n = 12); closed squares, BDL with 1 mg/kg LPS administration (n = 18); opened squares, sham-operated with 1 mg/kg LPS administration (n = 14). The survival of the groups was analyzed by Kaplan-Meier survival curves and log rank (Mantel-Cox) test. *P*<0.005 for BDL with 0.5 mg/kg and 1 mg/kg LPS administration vs. BDL group; *P*<0.05 for sham-operated rats with 1 mg/kg LPS administration vs. sham-operated group; BDL with 0.5 mg/kg vs 1 mg/kg LPS administration (log-rank test).

Death rates were compared between BDL and sham-operated rats. Fatality occurred most often within 4 hours of LPS administration. In both the BDL (n = 12) and sham-operated (n = 10) groups not given LPS, survival rates were 100%. In groups given LPS, the 24 h mortality rates were higher in the BDL group than in the sham-operated group at 0.5 mg/kg LPS (n = 24, 50% vs n = 12, 16.7%) and at 1 mg/kg LPS (n = 18, 90% vs n = 14; 50%) (Figure 6). In spite of this trend, statistical analysis did not show a significant difference between mortality rates for the groups at either 0.5 mg/kg LPS administration (*P* = 0.182) or 1.0 mg/kg LPS administration (*P* = 0.074). However, in the BDL groups, as compared with animals without LPS treatment, the 24 h mortality rates were significantly higher after 0.5 mg/kg (*P* = 0.004) and 1 mg/kg (*P*<0.001) of LPS; but in the sham-operated group, the mortality rate was significantly increased only after 1 mg/kg LPS (*P* = 0.011) (Figure 6). Endotoxin sensitivity seems to be higher in BDL than in sham-operated rats.

### Effects of LPS on CD14 protein expression in liver tissues

**Figure 7:**
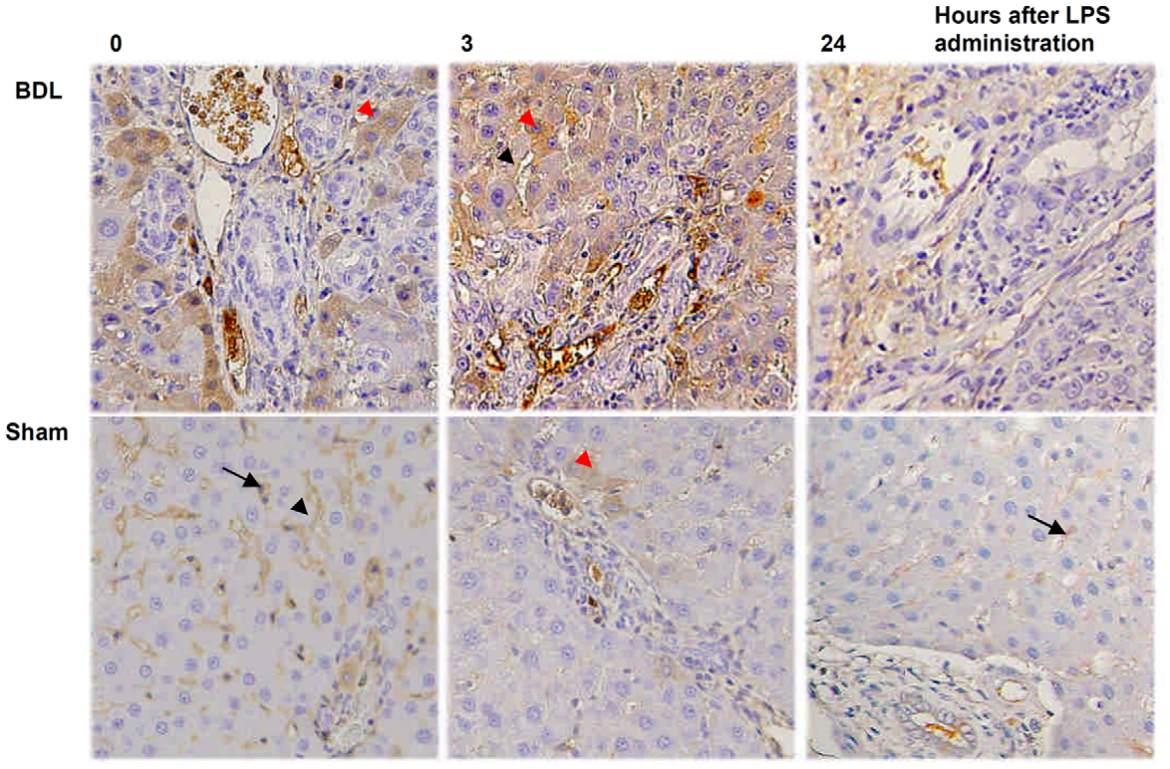
CD14 expression rat liver tissue after LPS administration. Comparison of CD14 expression in paraffin-embedded liver tissue sections among BDL and sham-operated rats after 0.5 mg/kg administration for 3 h and 24 h. Liver sections were stained with a polyclonal antibody against CD14 (dark brown) and counterstained with hematoxylin. CD14 immunoreactivity was detected in Kupffer cells (black arrow), sinusoidal endothelial cells (black arrowhead), and hepatocytes (red arrowhead). Original magnification: ×200.

Paraffin-embedded liver sections from rats were analyzed for CD14 localization using immunohistochemical staining. CD14 was observed in the parenchyma of the hepatic lobules, where Kupffer cells and sinusoidal endothelial cells were immunostained positive and the arterial and venous endothelium were immunostained negative (Figure 7). The expression of CD14 in the liver tissue of the BDL group was higher than in the sham-operated group, especially in the hepatocytes (Figure 7). Hepatocyte CD14 expression was significantly increased at 3 h after LPS administration in BDL rats, but the expression was markedly decreased at 24 h after LPS administration (Figure 7). However, the CD14 expression in the sham-operated group showed little change after the administration of LPS (Figure 7). Quantitative evaluation of CD14 positive cells in live tissues was performed by two experienced hepatopathologists. CD14 was considered to be activated if over 20% of the cells were immunochemically stained positive [3]. As shown in Table 2, CD14 activation ratios were significantly increased in rats receiving mock doses and 0.5 mg/kg LPS. These ratios remained high for 3 h after the termination of treatment in BDL relative to sham-operated groups.

**Table 2:** Indexes e of rat liver tissues with positive reaction.

	Indexes			
	CD14 activation		Endotoxin	
Time after 0.5 mg/kg LPS administration	Sham-operated	BDL	Sham-operated	BDL
	N (%)*	N (%)	N (%)	N (%)
0 h (n = 6)	0	3 (50)†	0	3 (50)†
3 h (n = 6)	3 (50)	6 (100)†	6 (100)	6 (100)
24 h (n = 6)	0	2 (33.3)	2 (33.3)	3 (50)

* Immunohistochemical CD14 or endotoxin staining in the liver tissues of rat among sham and common bile duct ligation group. The positive cells were >20% as positive.

† *p<0.05*, versus values for BDL compared with Sham-operated rats with Chi-square tests.

### Endotoxin levels and distribution in experimental rats

**Figure 8:**
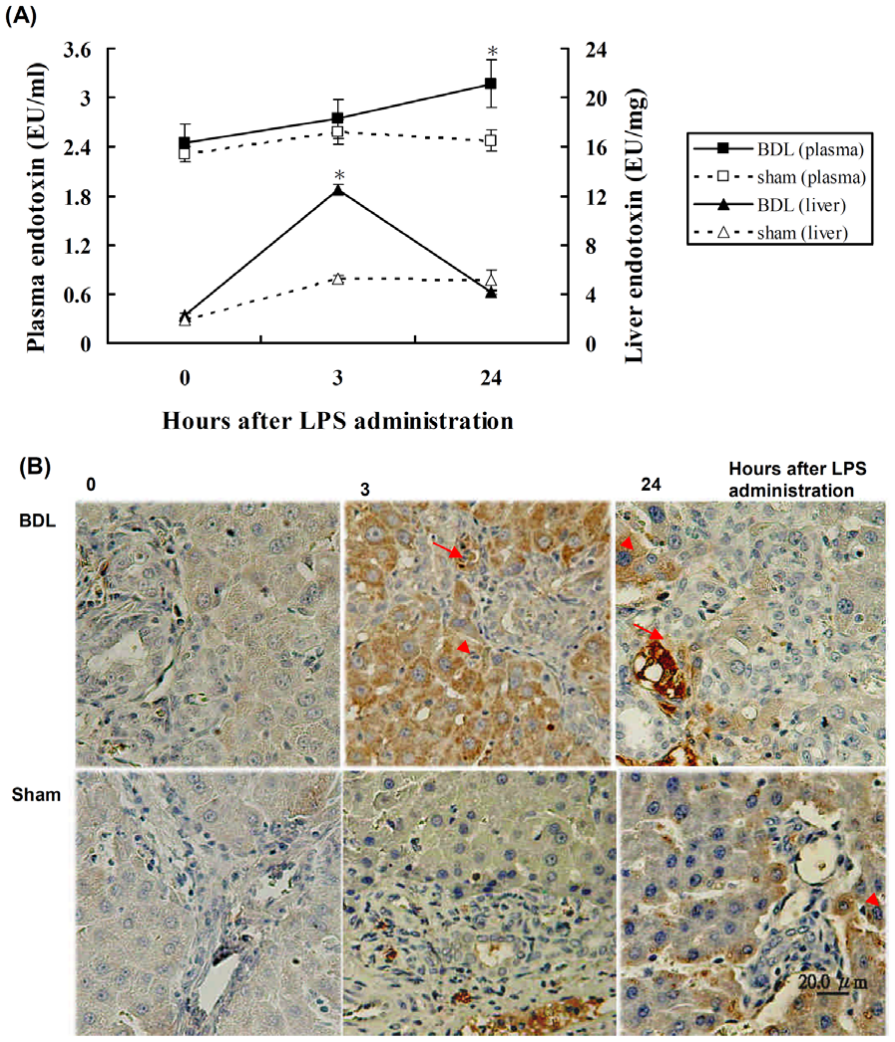
Endotoxin levels in plasma and liver tissues after administration of LPS. Time course of endotoxin levels in liver (circle) and plasma (square) of BDL (closed) or sham operation (open). (A) Endotoxin was assayed using polychrome LAL kits.* *P*<0.005 (Sham vs BDL group). (B) Immunohistochemical staining for endotoxin in paraffin-embedded liver tissue sections among BDL and sham-operated rats after 0.5 mg/kg administration for 3 h and 24 h. Liver sections were stained using a monoclonal antibody against lipid A (HM2046) and counterstained with hematoxylin. Lipid-A immunoreactivity was detected in hepatocytes (arrowhead) and biliary epithelial cells (arrow), Original magnification: ×200.

Plasma and liver endotoxin levels were assayed by LAL testing. Plasma endotoxin levels were not significantly different between the BDL and sham-operated animals (2.3±0.1 vs. 2.4±0.2 EU/mL, respectively, *P* = 0.7), but the endotoxin levels in liver tissues were higher in BDL rats than in sham-operated rats (2.7±0.3 vs 1.9±0.2 EU/mL, respectively, *P* = 0.03). Plasma endotoxin levels were significantly higher in the BDL group than in the sham-operated group 24 h after the administration of LPS (3.2±0.3 vs. 2.5±0.1 EU/mL, respectively, *P* = 0.002). After administration LPS, liver endotoxin levels were increased in both the BDL and sham-operated groups, particularly at 3 h after administration, when the BDL group showed significantly higher endotoxin levels than the sham-operated group (12.5±0.4 vs. 5.2±0.3 EU/mg, respectively, *P* = 0.001). At 24 h after LPS administration, there were no significant differences between the BDL (4.2±0.2 EU/mg) and sham-operated groups (5.2±0.9 EU/mg, *P* = 0.4) (Figure 8A).

Immunohistochemical staining using a monoclonal antibody against lipid A (Figure 8B) demonstrated weak or absent immunoreactivity in the liver tissues of sham-operated animals (Figure 8B). Strong lipid-A immunoreactivity was detected around the portal area in rat hepatocytes 3 h after the administration of LPS. This immunoreactivity was decreased in BDL-challenged animals at 24 h after the administration of LPS (Figure 8B). These findings are consistent with hepatic endotoxin levels. Quantitative evaluation of endotoxin-positive cells in live tissues was performed. As shown in Table 2, endotoxin accumulation was significantly higher in BDL than in sham-operated groups.

## Discussion

The role of CD14 as a key LPS signaling molecule has been well documented *in vitro* in many cell systems [12]–[14], [19]. Previous reports have shown that LPS modulates the expression of mCD14 in monocytes and macrophages [13]. Our data and those of others have indicated that hepatocytes express CD14 under basal conditions and that hepatic CD14 mRNA and protein levels are markedly increased during endotoxemia [3], [18], [20]. We therefore propose that hepatocytes contribute to both the basal systemic levels of sCD14 by contributing to the upregulation of CD14 that occurs during biliary atresia. However, the factors that govern both basal and inducible CD14 expression in liver cells are not well defined. In this study, we tested the hypothesis that LPS directly activates liver cells, leading to up-regulation of CD14 expression. We focused on the ability of LPS-elicited CD14 fluctuations in hepatocytes and HSCs during cholestasis to affect LPS reactivity and clearance. Our results showed significant enhancement in CD14 and MD2 expression after LPS treatment in C9 rat hepatocytes but not in rat hepatic stellate cells. Similarly, CD14 −376 promoter activity was enhanced after LPS treatment in C9 rat hepatocytes. This suggests distinct transcriptional activities and provides a basis for cell-specific regulation. It has been previously reported that CD14 transcription rates are significantly increased in the hepatocytes of LPS-treated rats [21], [22]. It has been indicated that the upregulation of CD14 mRNA levels observed in rat hepatocytes after LPS treatment is dose-dependent. Our data confirmed that hepatocytes *in vitro* exhibited an upregulated expression of CD14 mRNA and protein during the early phase of LPS challenge (1 h to 6 h) for promoting endotoxin clearance. In addition, MD2 mRNA and protein levels increased during the early phase of LPS challenge, inducing LPS-signal activation to produce proinflammatory cytokines as shown *in vivo* rat experiments (Figure 5).

The precise role of CD14 in LPS-induced signal transduction and LPS uptake is not yet clear. Both mCD14 and sCD14 may participate in the pathophysiology of endotoxemia and sepsis [12], [23]–[25]. Kupffer cells have been found to modify the endocytosed LPS and pass it on to hepatocytes, which subsequently excrete these products into the bile [26], [27]. Part of the LPS, though, is removed from the circulation directly by hepatocytes [28]. Although marked increases in CD14 mRNA caused by LPS stimulation have been observed *in vivo*, only modest increases in CD14 mRNA levels and promoter induction are caused by exposure to LPS in rat hepatoma cells [20]. In our previous report, CD14 was found to be expressed in an LPS-inducible manner in Kupffer cells, neutrophils, hepatocytes, and bile duct epithelia, suggesting a possible role for CD14 in hepatocytes during the uptake and clearance of LPS from the circulation. This is consistent with the notion that the synthesis of CD14 is part of an early-alarm system aimed at recognizing and binding LPS, enhancing the ability of the immune system to combat invading gram-negative bacteria. Early on during LPS challenge, the MD2 mRNA and protein levels became increased. The LPS-signal activation was then enhanced, leading to increased production of proinflammatory cytokines. These results are consistent with *in vivo* rat experiments. The remarkable elevation of CD14 in BDL animals early during LPS challenge suggests that CD14 probably acts as an early-phase response protein, which may indirectly increase endotoxin sensitivity and lead to host proinflammatory reactions in BDL rats.

Immunohistochemical analysis shows higher CD14 expression in Kupffer cells and sinusoidal endothelial cells in BDL rats (Figure 7). But the phagocytic function of Kupffer cells is impaired in cholestasis, and portal-derived endotoxin may accumulate in the liver and transfer into the peripheral circulation from the intestine [29]–[31]. CD14 production by hepatocytes and bile duct epithelial cells during cholestasis and the relationship with CD14 expressed on the Kupffer cells and sinusoidal endothelial cells is still not clear. It is suspected that the high levels of CD14 expression observed in Kupffer cells and sinusoidal endothelial cells may increase pro-inflammatory responses and cause cholestatic liver injury or lead to increased endotoxin-induced mortality [32]. Our data here show that BDL itself significantly increased serum levels of AST, ALT, and total bilirubin at 2 weeks after BDL, and these levels were all notably enhanced after biliary administration of LPS in BDL rats. LPS at doses of 0.5 mg/kg and 1 mg/kg resulted in a higher mortality rate for BDL animals (50% and 90%, respectively) than sham-operated animals (16.7% and 50%, respectively). These findings highlight the impact of cholestasis on the vulnerability of the rats towards endotoxemia. The initial hepatic responses to LPS occur in the hepatic microvasculature, including increases in leukocyte adhesion, reduction of sinusoidal perfusion, and activated Kupffer cells [33], [34]. Then, pro-inflammatory cytokines released from activated Kupffer cells, including tumor necrosis factor α (TNFα), are involved in the hepatic microvascular dysfunction [35], [36]. In this study, circulating TNFα was significantly enhanced after administration of LPS lasting 3 h in BDL rats. In contrast, monocyte chemoattractant protein-1 (MCP-1), which can cause Kupffer cells and neutrophils to release reactive oxygen species and toxicity in liver cells, increased in both BDL and sham-operated rats (Figure 5). Our experimental data support the conclusion that biliary obstruction enhances the inflammatory and microvascular responses of the liver after LPS challenge. Hepatocytes are the major source of most acute-phase proteins, including CD14, which is part of an adaptive response to tissue injury and infection under the control of LPS-signal activated cytokines.

Cholestatic liver disease ultimately leads to fibrosis because of hepatic reticuloendothelial system dysfunction and a hypersensitivity to endotoxin or bacterial challenge [2], [37]. The present study showed, by immunohistochemical staining and LAL assay, that basal hepatic endogenous endotoxin levels were higher in the BDL rats than in their sham-operated counterparts. Significantly more pronounced endotoxin accumulation was observed in hepatocytes after BDL. LPS challenge further aggravated the hepatic endotoxin levels in BDL animals compared with sham-operated animals as assessed 3 h but not 24 h after challenge. There was no significant difference in plasma endotoxin levels among any of the groups of animals at 3 h after administration of LPS, but endotoxin levels were notably increased in BDL animals relative to their sham-operated counterparts at 24 h. Possible explanations may be the systemic activation of macrophages in obstructive jaundice or a spillover of endotoxin from the portal to systemic circulation due to decreased clearance through the bile canalicular system [38]. In the present study, the effect of LPS on CD14 expression in liver tissue was evaluated using an experimental BDL rat model. CD14 was observed mainly on Kupffer cells and sinusoidal endothelial cells in the liver of normal or sham-operated rats. Its expression was mainly observed in hepatocytes 2 weeks after BDL and found to be especially increased 3 h after administration of LPS. However, CD14 expression was markedly decreased, down to basal levels, in both the BDL and sham-operated groups at 24 h after administration of LPS. Our findings suggest that LPS-induced CD14 expression behaved like an acute-phase protein, decreasing 24 hours after LPS challenge in a cholestatic animal model.

### Conclusion

The results of the present study suggest an enhanced vulnerability to LPS in a rodent model of obstructive jaundice. In sham-operated animals, enhanced hepatic CD14 expression was observed mainly in Kupffer and sinusoidal endothelial cells after LPS treatment. In the BDL animals, upregulated expression of CD14 in the BDL animals was observed mainly in hepatocytes after LPS challenge. These findings imply a different role for CD14 under normal and cholestatic conditions in sepsis. The remarkable elevation of CD14 observed in BDL animals early during LPS challenge suggests its possible nature as an early-phase response protein not only to clear the endotoxin but also to induce proinflammatory response. *In vitro* study further supports hepatocyte-specific CD14 transcriptional activities after LPS treatment, highlighting a possible role of hepatocyte-derived CD14 in endotoxemia. In summary, our *in vitro* and *in vivo* data indicate an enhanced sensitivity of hepatocytes to endotoxin with increased CD14 and MD2 expression during cholestasis. This may lead to proinflammatory reaction and cause lethal organ failure.

## Materials and Methods

### Cell culture

C9 rat liver epithelial cells were obtained from the American Type Culture Collection (ATCC, Manassas, VA, U.S.). The HSC-T6 cell line, immortalized rat HSCs transfected with the large T-antigen of SV40 vector containing a Rous sarcoma virus promoter, were maintained at 37°C in 5% CO_2_ and in Ham's F12 and Waymouth media (Invitrogen, Carlsbad, CA, U.S.), respectively and supplemented with 10% heat-inactivated fetal calf serum, 100 U/ml penicillin, and 100 mg/ml streptomycin [39]. The cells (5×10^6^) in 10 cm culture dishes were treated with various concentrations of LPS (1–1000 ng/mL) and harvested at different time intervals for Western blot and qRT-PCR analysis. LPS (L4391, *Escherichia coli*, 0111:B4) was purchased from the Sigma Chemical Company (St. Louis, MO, U.S.).

### Western blot analysis

The cells were scraped from the plate into protein extraction reagent (Pierce Chemical, Rockford, IL, U.S.). The lysates were centrifuged at 14,000 g for 15 min after the addition of protease inhibitors (0.5 mmole EGTA, 1 mmole/L PMSF, 1 mmole/L DTT, 25 µg/mL leupeptin, 25 mmole/L NaF, 1 mmole/L Na3VO4) and the supernatants were collected for Western blot analysis. The crude proteins were quantified with a Bio-Rad protein assay kit (Bio-Rad, Hercules, CA). The supernatants containing 30 µg crude proteins were treated with sample buffer (6% SDS, 1.4 M β-mercaptoethanol, 20% glycerol, 0.01% w/v bromphenol blue, and 125 mM Tris-HCl, pH 6.8), boiled for 10 min, separated on 10% SDS-PAGE gels, and transferred to polyvinylidene fluoride membrane (Millipore, Billerica, MA, U.S.). Membranes were blocked with 5% nonfat dried milk in Tris buffered saline buffer (TBS; 50 mM Tris-HCl, pH 7.4 and 150 mM NaCl) containing 0.1% Tween 20 (TBST) and incubated overnight with the following primary antibodies: anti-CD14 (M305; sc-9150;1∶3000; Santa Cruz Biotechnology, Santa Cruz, CA, U.S.), anti-TLR4 (25; sc-293072; 1∶3000; Santa Cruz Biotechnology, Santa Cruz, CA, U.S.), anti-MD2 (ab2418; 1∶2000; Abcam, Cambridge, MA, U.S.) and anti-β-actin (AC-15; A5441; 1∶3000; Sigma-Aldrich Company, S.t Louis, MO, U.S.). After washing with TBST, blots were incubated with HRP-conjugated secondary antibodies: anti-mouse IgG (ab6728; 1∶5,000; Abcam, Cambridge, MA, U.S.) for TLR4 and β-actin, anti-rabbit IgG (ab6013;1∶8,000; Abcam, Cambridge, MA, U.S.) for CD14 and MD2. Detection was achieved using a chemiluminescence substrate (Millipore, Billerica, MA, U.S.), and exposure to film. Signals were quantified by densitometric analysis.

### Real-time quantitative reverse transcription-polymerase chain reaction (qRT-PCR)

Total RNA was extracted from the harvested cells using TRIzol (Invitrogen, Carlsbad, CA, U.S.) according to the manufacturer's manual. A total of 2 µg of RNA was added to 0.1 µg of oligo-d (T)15 following the protocol of SuperScript®RT (Invitrogen, Carlsbad, CA, U.S.) for cDNA preparation. Quantitative PCR was performed in a final volume of 20 µl SYBR Green PCR mixture (Applied Biosystems, Foster City, CA, U.S.), and each sample was analyzed in duplicate. Each reaction mixture contained 0.2 pmole/µL of each primer, 1× SYBR Green PCR Master Mix, and 1–5 ng of cDNA. Thermal cycling was initiated with a 2 min incubation at 50°C, followed by a 10 min denaturation step at 95°C, and then 40 cycles of PCR consisting of 95°C for 15 seconds and 60°C for 1 min. GAPDH was used as an internal control for analysis of CD14, TLR4, and MD2 mRNA levels. The sequences of the PCR primers (Table S1) were designed based on cDNA sequences from GenBank. Quantification of the mRNA was achieved with an ABI PRISM 7700 Sequence Detection System (Applied Biosystems, Warrington, WA, U.S.) using comparative methods. The Ct values of CD14 were normalized to the Ct values of the housekeeping gene GAPDH.

### Construction of CD14 promoter and luciferase reporter gene assays

The region −1139 to +80 relative to the transcription start site from the CD14 promoter was PCR amplified from the genomic DNA of THP-1cells. The PCR product was digested by *Xho*I and *Hind*III enzymes and then subcloned into pGL3-Basic vector (Promega, Madison, WI, U.S.). Unidirectional constructs of pGL3-CD14-1139 were prepared according to the predicted map of CD14 (Fig. 3A) by direct PCR using specific primers (Table S2). Constructs of deletion clones were verified by sequence analysis and were prepared using the endotoxin-free midiprep kit (Promega, Madison, WI, U.S.) for transfection.

Plasmid DNA of CD14 promoters with luciferase vector and pRL-TK (Promega, Madison, WI, U.S.) as internal control were transfected into cells using Lipofectamine 2000 (Invitrogen, Carlsbad, CA, U.S.). After transfection for 12–16 h, cells were washed twice with Hank's balanced salt solution and cultured with ITSA medium (Ham's F-12/DMEM (1∶1) containing 1X Insulin-Transferrin-Selenium-A and 0.1% BSA) for 24 h. The cells were cultured for the indicated times and lysed with 1× passive lysis buffer (Promega, Madison, WI, U.S.). The luciferase activities were obtained using an EG&G Berthold Microplate Luminometer (LB 96 V; Berthold Technologies, Germany). The relative activity was calculated as a ratio of CD14 promoter-firefly luciferase and TK-renilla luciferase.

### *In vivo* animal experiments

All animal experiments were performed in accordance with and were approved by the Animal Care and Use Committee of Kaohsiung Chang Gung Memorial Hospital. Male Sprague-Dawley rats (weighing 300–330 g) were divided into two groups. After anesthesia with intraperitoneal injection of thiopentone sodium (50 mg/kg Pentothal; Abbott Laboratories, Chicago, IL, U.S.), each rat underwent laparotomy and a silicone catheter (Silicone Elastomer; Helix Medical, Carpinteria, CA, U.S.) with an inside diameter of 0.508 mm and outside diameter of 0.930 mm was inserted into the proximal and distal bile ducts with the middle segment tunneled and located in the subcutaneous space. The procedure was performed as previously described [40]. Rats subjected to ligation of the subcutaneous segment of the indwelling catheter, which caused complete biliary obstruction, were defined as bile duct-ligated (BDL) rats. Rats that did not undergo ligation of the catheter served as sham-operated controls. Two weeks after surgery, rats in both groups were disinfected under anesthesia, and a small incision was made over the previous laparotomy wound. LPS dissolved in PBS buffer (0.5 mg/kg or 1 mg/kg body weight) was injection into the biliary system through the indwelling silicone catheter. The rats were further divided into those killed 3 h and those killed 24 h after LPS treatment. Blood samples were collected before animal euthanasia. Serum aspartate aminotransferase (AST), alanine aminotransferase (ALT), and total bilirubin (T-Bil) levels were determined using a biochemistry auto-analyzer (Model 7450; Hitachi, Tokyo, Japan). Liver tissues were fixed in 4% paraformaldehyde and embedded in paraffin for immunohistochemical analysis. The normal control group received neither the operation nor the LPS treatment.

### Assay for TNFα and MCP-1

Plasma levels of TNFα and MCP-1 were determined using enzyme linked immunosorbent assay (ELISA) kits (rat TNFα and rat MCP-l; Biosource, Camarillo, CA, U.S.) according to the manufacturer's guidelines. All experiments were performed in duplicate.

### Limulus amebocyte lysate (LAL) test

Plasma specimens were collected aseptically in nonpyrogenic containers. The plasma and liver specimens were diluted 1∶10 and assayed for endotoxin with a commercially available pyrochrome LAL kit (Associates of Cape Cod, Falmouth, MA, U.S.) according to the manufacturer's instructions.

### Immunohistochemical staining for CD14 and lipid A

Immunoreactive CD14 and lipid-A stainings were performed on rat liver tissue samples that had been formalin-fixed and paraffin-embedded. Two-micrometer sections were deparaffinized, treated with 3% hydrogen peroxide to inactivate the endogenous peroxidase activity, and microwaved for 7 min in 10 mM citrate buffer (pH 6.0) to retrieve the antigen. The sections were then incubated in PBS supplemented with 5% fetal calf serum for 10 min to block background interactions. The sections were then incubated with rabbit anti-CD14 antibody (1∶200; SC-9150; Santa Cruz Biotechnology, Santa Cruz, CA, U.S.) or a monoclonal anti-lipid A antibody (1∶100; 43; HM2046; HyCult Biotechnology, the Netherlands) at 37°C for 2 h. Negative controls for immunohistochemical staining using normal rabbit IgG (1∶200; SC -2027, Santa Cruz Biotechnology, Santa Cruz, CA, U.S.) instead of primary polyclonal antibody against CD14 and mouse IgG1 isotype control antibody (ab27479; Abcam, Cambridge, MA, U.S.) instead of primary monoclonal antibody against lipid A were included to indicate the extent of any nonspecific binding [3]. The sections were washed with PBS supplemented with 0.05% Tween 20 and then incubated for 10 min with the secondary antibodies (SuperPicture; Zymed Laboratories, Francisco, CA, U.S.). DAB color substrate (DAKO, Carpinteria, CA, U.S.) was added to cover each section, and the reaction was stopped with ddH_2_O. The slides were counterstained with hematoxylin, and mounted in mounting medium. Quantitative evaluation of CD14 and endotoxin-positive cells in liver tissues was performed by two experienced hepatopathologists. CD14 or endotoxin was considered to be activated if positive cells covered over 20% of the tissue area. Positive indexes in the liver sections of BDL and sham-operated groups were calculated.

### Statistical analysis

All statistical analyses were performed using the SPSS statistical software package, version 16 (SPSS, Chicago, IL, U.S.). Data are presented as mean ± standard error. Differences were evaluated using analyses of variance followed by Bonferroni's test or t-tests where appropriate. Correlations were examined with two-tailed Pearson correlation analysis. Differences in mortality between groups were determined using Kaplan-Meier survival curves and log rank (Mantel-Cox) test. *P*-values of less than 0.05 were considered significant.

## Supporting Information

Table S1 Primer sequences used for qRT-PCR detection of expression.
(DOC)

Table S2 Primer sequences for CD14 promoter reporter gene constructions.
(DOC)
