# Supplementary material for: Effects of Hepatocyte CD14 Upregulation during Cholestasis on Endotoxin Sensitivity
Source: PLoS One. 2012 Apr 12;7(4):e34903. doi: 10.1371/journal.pone.0034903 (PMC3325271; doi:10.1371/journal.pone.0034903)
Supplement: Table S1 — Primer sequences used for qRT-PCR detection of expression. (DOC) [file pone.0034903.s001.doc]

**Table S1. Primer sequences used for qRT-PCR detection of expression**

|  | Forward (5′–3′) | Reverse (5′–3′) |
| --- | --- | --- |
| CD14 | ACT GAC TCT TGA AAA CCT CG | AGC GCT AAA ACT TGG AGG GT |
| TLR4 | CTC GAG CCA GAA TGA GGA CTG | AGC GGC TAC TCA GAA ACT GC |
| MD2 | ACG CTG CTT TCT CCC ATA | CAC AAA TCC ATT GGT TCC CT |
| GAPDH | TCA CCA CCA TGG AGA AGG C | GCT AAG CAG TTG GTG GTG CA |
