# Supplementary material for: Effects of Hepatocyte CD14 Upregulation during Cholestasis on Endotoxin Sensitivity
Source: PLoS One. 2012 Apr 12;7(4):e34903. doi: 10.1371/journal.pone.0034903 (PMC3325271; doi:10.1371/journal.pone.0034903)
Supplement: Table S2 — Primer sequences for CD14 promoter reporter gene constructions. (DOC) [file pone.0034903.s002.doc]

Table S2. Primer sequences for CD14 promoter reporter gene constructions

| Name Sequences |
| --- |
| CD14-1139F GGGGTACCCCAGGAGAGAGCAACGTGCAAG |
| CD14-449F GGGGTACCCCAGGGACTTGGATTTGGTGGC |
| CD14-376F GGGGTACCCCGATTCTCTGGGATATAAGGT |
| CD14-300F GGGGTACCCCATCATCCTTTTCCCACACCC |
| CD14-232F GGGGTACCCCGGCGCCTGAGTCATCAGG |
| CD14-+80R CCGCTCGAGCGGTCTGAGCTCCGGACAG |
